# Combination of neutrophil/lymphocyte ratio and albumin/bilirubin grade as a prognostic predictor for hepatocellular carcinoma patients undergoing curative hepatectomy

**DOI:** 10.1186/s12876-023-02804-5

**Published:** 2023-05-19

**Authors:** Hao Luo, Chongming Huang, Meng Meng, Ming Zhang, Zigang Li, Jun Huang

**Affiliations:** 1 Department of General Surgery, The First People’s Hospital of Yibin, Yibin, Sichuan 644000 PR China; 2 Department of Gastrointestinal Surgery, Shandong Provincial Third Hospital, Shandong University, Jinan, 250031 PR China

**Keywords:** Hepatocellular carcinoma, Prognostic factor, Neutrophil/lymphocyte ratio, Albumin/Bilirubin grade, Overall survival

## Abstract

**Background and aim:**

Prognosis determination is essential for hepatocellular carcinoma (HCC) patient management and treatment planning. The current study aimed to evaluate the prognosis performance of NLR, ALBI, and the combination of NLR-ALBI in determining the overall survival (OS) of HCC patients under curative hepatectomy.

**Methods:**

144 primary HCC patients with curative hepatectomy were recruited in the retrospective study. The clinicopathologic characteristics and OS were compared between stratified groups. The predictive performance of NLR, ALBI, and the combination of NLR-ALBI was explored by the area under the receiver operating characteristic curve (AUC). Univariate and multivariate analyses were used to determine the risk factors of OS.

**Results:**

AUC determined NLR cutoff > 2.60 for predicting prognosis. The univariate analysis indicated pathological differentiation, tumor size, AFP, TNM stage, NLR score, and ALBI grade were significant indicators of OS. However, only TMN grade, AFP, NLR score, and NLR-ALBI score were identified as independent predictors of OS in the multivariable analysis. The AUC of NLR, ALBI and the combination of NLR-ALBI was 0.618(95%CI 0.56–0.710), 0.533 (95%CI 0.437–0.629), 0.679 (95%CI 0.592–0.767) respectively. Patients with higher NLR-ALBI scores presented worse outcomes than those with lower NLR-ALBI scores.

**Conclusion:**

NLR is an independent prognostic factor of HCC and a reliable biomarker in predicting the OS of HCC patients. The combination of NLR-ALBI showed a better prognostic performance than using NLR or ALBI alone, implicating the effectiveness and feasibility of combining multiple risk factors for postoperative prognosis assessment.

## Introduction

Due to the burden of chronic HBV infection, China accounts for over 50% of all newly diagnosed liver cancer cases and deaths in the world, even though the total population in China is only 20% of the world [1–3]. Although the age-standardized incidence rates of liver cancer have declined in China recently [3–5], HCC still ranks as the second most common malignancy, with 360,00 incident cases reported per year, and the second cause of cancer death leading to 350,000 deaths per year in China [5–8]. More importantly, several studies have shown that the overall average medical expenditure and economic burden of HCC management have dramatically increased in China [9–11]. Hence, HCC imposes a significant economic burden on patients’ families and the health system.

Following a diagnosis of liver cancer, one immediate challenge in patient management is the determination of prognosis. Over the last several decades, many traditional clinicopathological prognostic factors and serum biomarkers have been widely applied to determine the prognosis. These score systems or strategies included, but were not limited to, serum alpha-fetoprotein (AFP), Child Turcotte Pugh (CTP), Barcelona Clinic Liver Cancer (BCLC), the Japanese Tumor Node Metastasis staging system, etc. These staging systems have been approved to be useful but are also limited by their complexity and subjectivity. For example, CTP scoring includes ascites and encephalopathy, subject to inter-observer variation [12], and serum AFP is not sensitive in patients with small tumors. Recently, two simpler prognostic markers: Neutrophil to Lymphocyte Ratio (NLR) and the Albumin Bilirubin (ALBI) grade, are considered emerging prognostic indicators in HCC.

Both systematic and tumor micro-environmental inflammation contribute to cancer development and progression. NLR is one of the generic and straightforward biomarkers to indicate cellular immune response under different disease conditions. Hence, it is not surprising that emerging evidence has shown that NLR could be a useful prognostic index in many liver diseases, including HCC. Several meta-analyses have systematically evaluated the role of NLR as a prognostic biomarker in HCC [12–14]. These works have approved that NLR was a reliable biomarker with prognostic potential for HCC, independent of various treatments. For example, one meta-analysis with 54 studies reported that NLR performed better than the conventional alpha-fetoprotein in predicting HCC survival [12]. Similar findings have been reported in another meta-analysis encompassing 15 studies and 3094 HCC patients [13].

ALBI grade is another mathematical model created by Johnson in 2015 based on the data of 1313 Japanese patients [15], which can evaluate the liver function in patients with HCC. Unlike the CTP score, the ALBI grade only uses two simple objective parameters, enabling a better evaluation [16]. Since then, the use of ALBI grade in HCC has gained wide attention [15–20]. Gui and his colleagues found that ALBI grade demonstrated clear survival discrimination superior to CTP class in HCC patients with yttrium-90 radioembolization treatment [17]. Even for patients with early-stage HCC, ALBI grade was strongly associated with recurrence and long-term survival and was sensitive enough to define the outcome [18]. In a meta-analysis with 95 studies describing the relationship between HCC and ALBI, Bannaga and his colleagues reported that ALBI grade in HCC predicts survival better than alpha-fetoprotein and CTP score [12].

In the current study, we retrospectively analyzed clinical data and outcomes of 144 HCC patients from our center. We found that the preoperative NLR and ALBI grade had good predictive value for the postoperative prognosis of patients with HCC. However, multivariable analyses only indicated NLR, not ALBI grade, was an independent prognostic factor in our cohort. More importantly, the combination of the NLR-ALBI score confers better prognostic value than using NLR or ALBI grade alone, implicating the effectiveness and feasibility of combining multiple risk factors for postoperative prognosis assessment.

## Method

### Patient enrollment

Patients with primary hepatocellular carcinoma (HCC) from Jan/2013 to Jan/2017 were recruited from Hepatology Clinic at the first people’s Hospital of Yibin. The inclusion criteria were as follows: (1) diagnosed with primary HCC without metastasis by post-operation pathology; (2) all patients underwent curative hepatectomy; (3) All patients did not receive tumor-related treatments before curative hepatectomy; (4) with complete medical record and follow-up. Patients with hepatic tumors other than HCC, or HCC with metastasis, or died for non-HCC-associated reasons were excluded from the analysis. After screening, 144 patients were identified and recruited using the hospital informatics system.

The study was approved by the Institutional Review Board of the First People’s Hospital of Yibin (IRB 2023003). Because of the retrospective design of our study and only de-identified data was collected, the request of informed consent was waived by the Institutional Review Board. All methods were carried out in accordance with relevant institutional guidelines and regulations.

### Data collection and follow-up

Baseline clinical examination and tumor characteristics were collected and assessed retrospectively. The following information was extracted from patients’ medical records for analysis: age, sex, HBsAg positive, liver cirrhosis, blood chemistry, serum AFP level, pathological findings (tumor size, histological grade, number of tumors), and abdominal imaging at the time of HCC diagnosis. The tumor size was determined by either CT or MRI imaging at the diagnosis of HCC. The Albumin Bilirubin (ALBI) grade was calculated using the system described in 2015 by Johnson et al. [15], and Edmondson and Steiner criteria [21] were used to evaluate the histological differentiation of HCC. Child Turcotte Pugh (CTP) score was calculated based on the method created by Child CG and Pugh RN [22].

Patients were followed once every six months after surgery. The last day for the follow-up of the current study was Dec/31/2019, with an average following time of 31.6 ± 1.6 months, ranging from 1 to 69 months. Survival was defined as the time interval between the date of HCC diagnosis and the death or last follow-up examination.

### Statistical analysis

The data are presented as means ± standard deviations (SD) or percentages appropriately for the data type. A 2-tailed t-test or Chi-squared test was used to perform univariate analyses as appropriate. Then, these variables with a p-value < 0.05 on univariate analyses were further applied for the multivariate Cox proportional hazard regression analysis to determine hazard ratios and 95% confidence intervals associated with overall survival (OS) using the stepwise backward selection process. The risk levels of OS were defined based on the number of risk factors present, with Kaplan–Meier survival curves constructed for each risk level. Receiver operator characteristic (ROC) curves were used to determine optimal NLR cutoffs, and the cutoff points were selected by maximizing Youden’s index. All statistical analyses were conducted using GraphPad software (Version 5.01, GraphPad, CA, USA).

## Results

### Patient characteristics

One hundred and forty-four HCC patients were included in the retrospective studies, with 119 males (82.6%) and 25 females (17.4%). The average age of disease diagnosis was 53.2 ± 10.1 (ranging from 30 to 84 years). Among these patients, 110 (76.3%) were HBsAg positive, and 93 (64.5%) presented with liver cirrhosis at the time of diagnosis, 55 patients had serum AFP levels ≥ 400, and the median tumor size was 5.05 cm (ranging from 1.0 to 16.0 cm). For TMN grade, the number of cases in grade I, II, and III was 60 (41.7%), 37 (25.6%), and 47 (32.6%), respectively. At the end of the follow-up, 80 patients (55.6%) died during the three years follow-up period. The baseline demographic and clinicopathologic characteristics between survival and death groups were summarized in Table 1. As Table 1 shows, these patents from non-survival group had significant large tumor size (7.93 ± 3.33 *vs*. 4.55 ± 2.13 of survival group, *P* < 0.000), and increased NLR score (3.79 ± 3.29 *vs*. 2.80 ± 1.89 of survival group, *P* < 0.000). Patients from non-survival group also had advanced pathology evaluation, as evidenced by TMN and tumor differentiation grading (*P* < 0.001, and *P* < 0.003, respectively, compared to survival group).

**Table 1:** Clinicopathologic characteristics of HCC patients between survival and non-survival groups

	Survival group (n = 64)	Non-survival group (n = 80)	*P* value
Gender (M/F)	54/10	65/15	NS
Age (yrs)	54.5 ± 10.3	52.0 ± 9.85	NS
HBsAg (Y/N)	48/16	62/18	NS
Cirrhosis (Y/N)	44/20	49/31	NS
Tumor Size	4.55 ± 2.13	7.93 ± 3.33	0.000
Number of tumors (S/M)	59/5	67/13	NS
AFP ≥ 400 (Y/N)	12/52	43/37	0.0001
AST (U/L)	49.9 ± 35.0	52.0 ± 36.3	NS
ALT (U/L)	45.2 ± 21.4	56.2 ± 42.2	NS
Scr (µmol/L)	71.1 ± 14.8	71.3 ± 16.2	NS
Hospital duration	20.6 ± 7.03	21.4 ± 8.47	NS
Differentiation	21/43	46/34	0.003
Child-Pugh Score			
A	57	70	0.773
B	7	10	
TMN Grade (I/II/III)			
I	48	12	0.000
II	16	21	
III	0	47	
NLR	2.80 ± 1.89	3.79 ± 3.29	0.000
ALBI grade			
I	48	39	0.001
II	16	41	
NLR-ALBI			
Score 1	34	17	0.000
Score 2	24	34	
Score 3	8	27	

M: male; F: female; Y: yes; N: no; S: Single: M multiple; NS: no significant

### The association of the NLR-ALBI score with the clinicopathological features of HCC

To evaluate the prognostic performance of combing NLR score and ALBI grade (NLR-ALBI score), we first defined ALBI grade 1 and 2 as 0 and 1, respectively, and then scored NLR ≤ 2.60 and NLR > 2.60 as 0 and1, respectively. For each patient, we added the score of ALBI grading with the score of NLR together to get the combined value of the NLR-ALBI score. Based on the new score system, we categorized patients into three groups. Group A (NLR-ALBI score = 0), Group B (NLR-ALBI score = 1), and Group C (NLR-ALBI score = 2), as Table 2 illustrated.

**Table 2:** Relationship between NLR-ALBI grade and clinicopathological features of patients with hepatocellular carcinoma

Index		Groups			X^2^/ F value	*P* value
		Score = 0	Score = 1	Score = 2		
Age	< 60yrs	36	44	26	0.011	0.994
	≥ 60yrs	13	16	9		
HBsAg	Positive	9	19	6	3.712	0.156
	Negative	40	41	29		
Cirrhosis	Yes	11	28	12	6.942	0.031
	No	38	32	23		
Child-Pugh score	A	47	57	27	10.795	0.005
	B	2	3	8		
Differentiation	Low	10	22	13	4.066	0.131
	Medium to High	39	38	22		
AFP	< 400	36	36	17	5.504	0.064
	≥ 400	13	24	18		
Number of tumors	Single	43	56	27	5.302	0.071
	Multiple	6	4	8		
Tumor size (cm)	< 5 cm	30	15	4	26.286	< 0.001
	≥ 5 cm	19	45	31		
TNM grade	I	31	23	6	18.339	< 0.001
	II-III	18	37	29		
TBIL (µmol/L)		14.25 ± 7.45	13.96 ± 5.28	17.55 ± 7.03	3.761	0.026
ALB (g/L)		42.39 ± 2.74	41.04 ± 4.19	37.31 ± 3.01	22.659	< 0.001
PLT (S)		138.53 ± 69.34	165.07 ± 76.90	167.03 ± 101.64	1.865	0.165
ALT (U/L)		46.21 ± 25.99	50.46 ± 37.62	65.01 ± 37.31	4.459	0.013
AST (U/L)		47.06 ± 26.90	50.87 ± 41.71	57.07 ± 35.15	0.807	0.448
PT (x 10^9^)		12.79 ± 1.65	13.07 ± 1.43	13.63 ± 1.85	2.833	0.062
Scr (µmol/L)		73.16 ± 14.41	70.40 ± 16.29	69.90 ± 15.97	0.588	0.557
CEA (µmol/L)		2.07 ± 1.65	3.98 ± 13.07	2.20 ± 1.99	0.825	0.440
Hospital Duration (D)		18.57 ± 6.81	21.80 ± 8.62	23.31 ± 7.05	4.368	0.014

The clinicopathological features between these three groups are summarized in Table 2. A strong association between NLR-ALBI score and Liver cirrhosis (x^2^ = 6.942, *P* = 0.031), CTP score (x^2^ = 10.795, *P* = 0.005), tumor size (x^2^ = 26.286, *P*<0.001)were detected. Patients with higher NLR-ALBI score (Score = 1 or 2) presented with the lower serum ALB level(x^2^ = 22.659, *P *<0.001); higher serum ALT level (x^2^ = 4.459, *P* = 0.013), increased TNM grade (x^2^ = 18.339, *P *<0.001). These patients with higher NLR-ALBI scores also stayed at the hospital longer than those patients with low NLR-ALBI scores (hospital duration, x^2^ = 4.368, *P* = 0.014). Together, these results indicated that the NLR- ALBI score would reflect the severity of HCC.

### The prognostic performance of the NLR score, ALBI grade, and the NLR-ALBI score

**Fig. 1:**
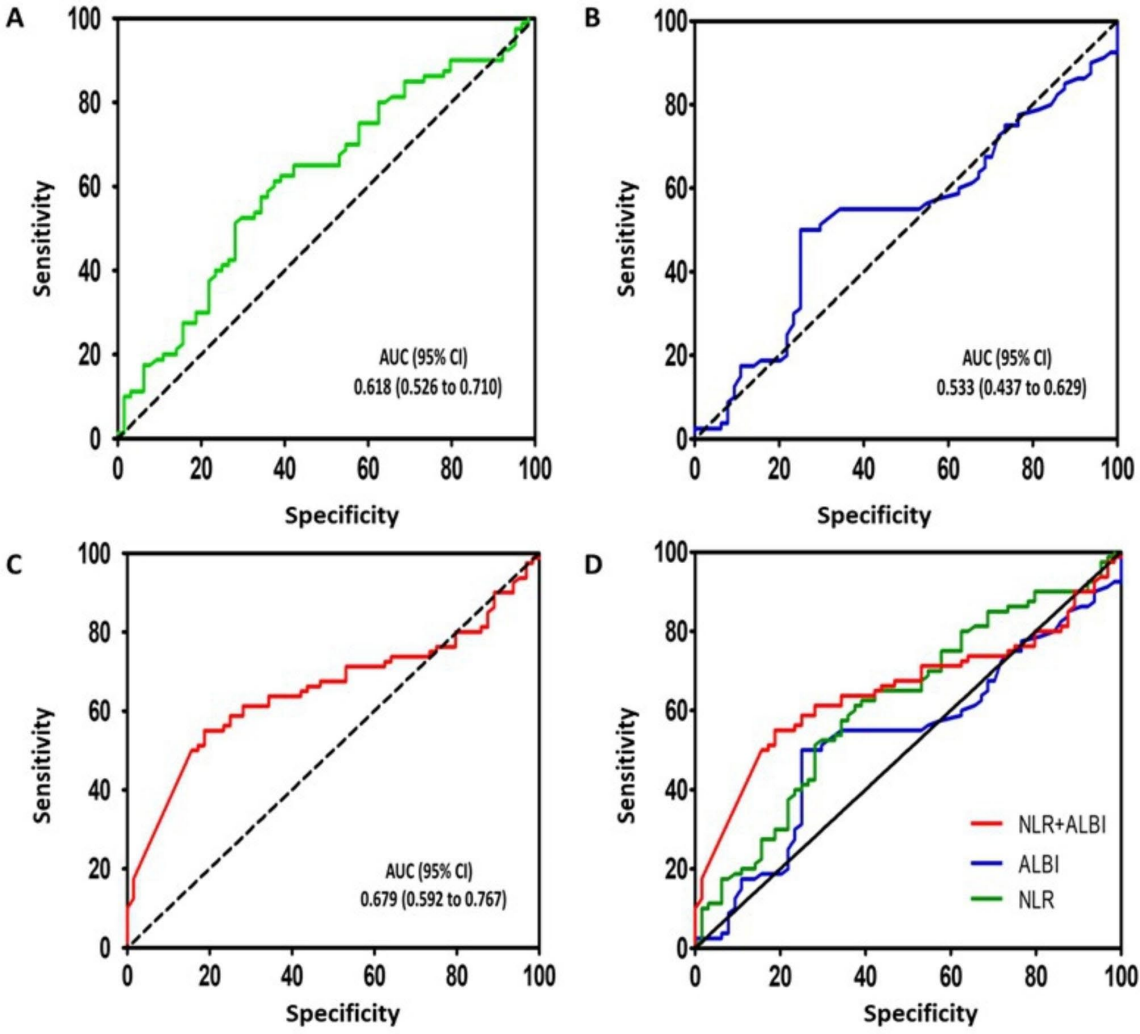
Receiver operating characteristic curves for pre-treatment NLR **(A)** and ALBI grade **(B)**, the combination of NLR-ALBI **(C)** in predicting overall survival of HCC patients underwent curative hepatectomy. The **(D)** illustrated the overall comparison among these three indicators. NLR: neutrophil to-lymphocyte ratio; ALBI Albumin/Bilirubin grade AUC: area under the curve; CI: confidence interval

Receiver operating characteristic (ROC) curves analysis was used to determine the prognostic performance of NLR, ALBI alone, or the combination of NLR-ALBI. The results are presented in Fig. 1. The AUC of NLR was 0.618, with 95%CI 0.56–0.710. When ALBI was used alone, it yielded an AUC value of 0.533, with 95%CI 0.437–0.629. The combined use of NLR and ALBI produced a larger AUC (0.679, 95%CI 0.592–0.767) than NLR or ALBI used alone.

### Kaplan-Meier curves analysis for OS of subgroup patients with NLR score, ALBI grade, and NLR-ALBI score

**Fig. 2:**
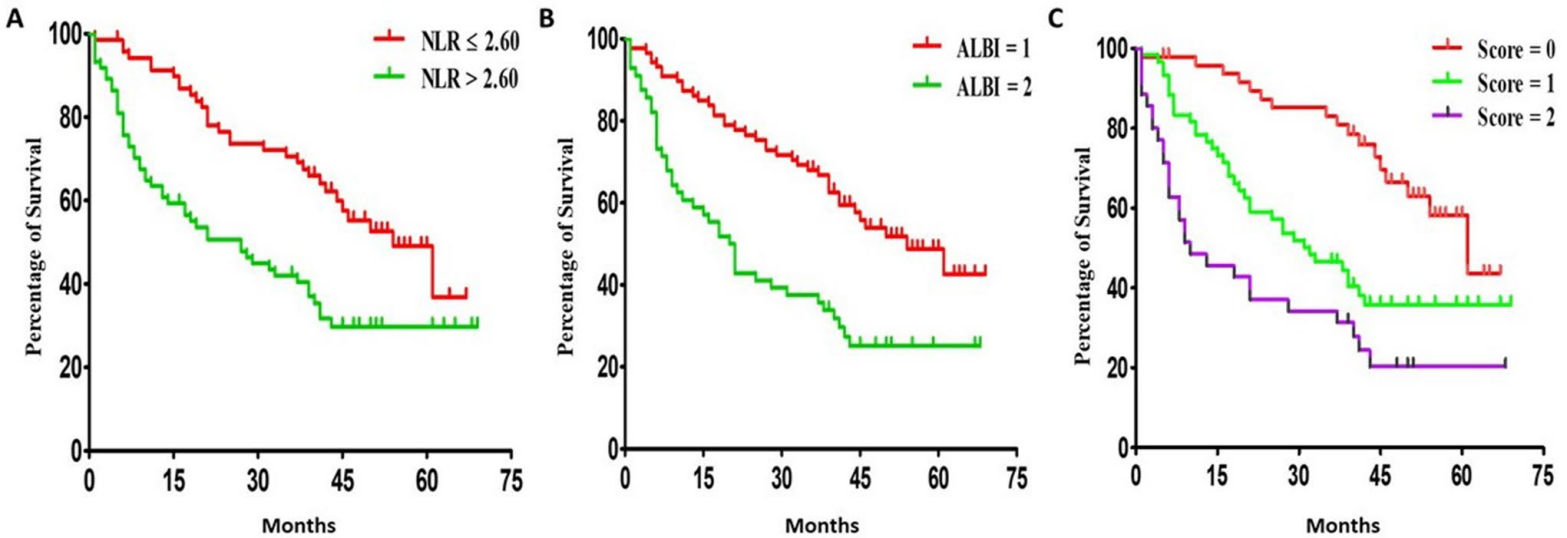
**(A)** Kaplan-Meier curves for Overall survival (OS) of patients with high NLR (> 2.60) and low NLR (≤ 2.60). **(B)** Kaplan-Meier curves for OS of patients with ALBI score 1 and ALBI score 2. **(C)** Kaplan-Meier curves for OS of patients with different NLR-ALBI scores. Censored events are represented by vertical lines. NLR: Neutrophil to-lymphocyte ratio; ALBI: Albumin/Bilirubin grade

In our cohort, the median and mean values of the NLR score were 2.64 and 3.34, respectively, with an SD of 2.79 and a range from 0.78 to 25.74. The ROC analysis showed that the optimal cut-off value of NLR was 2.60 (95% CI 0.582–0.760). Therefore, we divided patients into the low NLR group (≤ 2.60, n = 71) and high NLR group (> 2.60, n = 73). The one- and three-year OS was 67.2% and 32.9% for the high NLR group, respectively, while it was 91.4% and 63.0% for the low NLR group, respectively, with significant differences (log-rank test, x^2^ = 12.30, *P*<0.005), as demonstrated in Fig. 2A.

For ALBI grades, according to the Johnson’s criteria [15], patients were categorized into grade 1 (defined as ≤ − 2.60 score), grade 2 (score greater than − 2.60 to ≤-1.39), and grade 3 (score ≥ -1.39). In our cohort, there were 87 ALBI grade 1 patients and 57 grade 2 patients, but no patients sub-grouped into grade 3. Accordingly, as shown in Fig. 2B, the ALBI grade 2 patients had worse OS, with one-and 3- year OS rates of 67.7% and 38.6%, respectively, while the one- and 3-year OS for the grade 1 group were 87.2% and 61.4%, respectively, with significant difference (log-rank test, x^2^ = 16.10, *P*<0.001).

Kaplan-Meier curves analysis also demonstrated the significant OS difference among three NLR-ALBI subgroups. As Fig. 2C shows, patients with NLR-ALBI score = 0 had the best OS, with 1- and 3-year OS at 95.8% and 83.1% respectively, and patients with NLR-ALBI score = 1 presented with the 1 -and 3- year OS at 78.3% and 46.5%, respectively. However, the patients with the highest NLR-ALBI score (score = 2) had the worst outcome, with 1 -and 3- year OS at 48.6% and 34.4%, respectively, which was significantly lower than that of score = 0 and score = 1 patients (log-rank test, x^2^ = 24.09, *P*<0.001).

### Univariate and multivariate analyses of OS of HCC of our cohort

In our cohort, the median OS was 14.0 months (95% confidence interval [CI]: 10.4–17.6 months), and the 1-, 3-, and 5-year OS rates were 52.3%, 27.1%, and 23.3%, respectively. Pathological differentiation, tumor size, AFP, TNM stage, NLR score, and ALBI grade were identified as significant indicators of OS in the univariate analysis (Table 3). However, only TMN grade (hazard ratio [HR]: 5.853, 95% CI: 3.015–11.364; *P* < 0.001), AFP (HR: 2.366; 95% CI: 1.489–3.7589; *P* < 0.001), and NLR score (HR: 1.832, 95% CI: 1.157–2.901; *P* = 0.01). NLR-ALBI score (HR:1.973, 95% CI: 1.134–3.434; *P* = 0.016) were identified as independent predictors of OS in the multivariable analysis (Table 3). Notably, our multivariate model did not find the ALBI grade as an independent factor in predicting OS (*P* = 0.574).

**Table 3:** Univariable and multivariable analyses of overall survival (OS) in patients with hepatocellular Carcinoma

	Univariable analyses			Multivariable analyses		
	*X* ^*2*^	*P*		*HR*	95% *CI*	*P*
Gender	0.006	0.936				
Age (yr)	2.310	0.129				
HBsAg positive	0.966	0.326				
Liver cirrhosis	0.941	0.332				
AST	0.397	0.529				
Child-Pugh score	1.243	0.235				
Differentiation	15.240	< 0.001		0.882	0.539–1.448	0.619
Number of tumors	2.092	0.148				
Tumor size	40.274	< 0.001		1.800	0.774–4.176	0.171
TNM grade	58.004	< 0.001		5.853	3.015–11.364	< 0.001
AFP	31.219	< 0.001		2.366	1.489–3.759	< 0.001
NLR	14.162	< 0.001		1.832	1.157–2.901	0.010
ALBI grade	14.582	< 0.001		1.148	0.710–1.855	0.574
NLR-ALBI	25.028	< 0.001		1.973	1.134–3.434	0.016

## Discussion

Prognosis plays a critical role in patient management and decision-making, especially in the field of oncology. Determining relevant prognostic factors not only guides treatment planning but also helps us understand the pathogenesis and the natural course of the disease [23]. In the current work, we evaluated the prognostic value of pre-operative NLR score and ALBI grade in predicting the OS of HCC patients underwent curative hepatectomy from our center. Our work indicated NLR, not ALBI grade, was an independent prognostic factor in our cohort. We also found the combination of the NLR-ALBI score confers better prognostic value than using NLR or ALBI grade alone.

Inflammation plays a vital role in the development and progression of HCC [24, 25]. NLR, the ratio between neutrophil and lymphocyte counts, can reflect the potential balance between neutrophil-associated pro-tumor inflammation and lymphocyte-dependent anti-tumor immune function. As an indicator reflecting patients’ inflammatory status, NLR has been approved as a reliable index to predict the prognosis of various cancers, including HCC. In line with previous studies addressing the link of NLR with HCC [12–14, 26], our cohort also demonstrated that NLR was associated with tumor size and TNM grade. More importantly, as Fig. 2A shows, the median survival of patients with NLR ≤ 2.6 was 54 months, while it was 27 months for these patients with NLR > 2.60, indicating that patients with higher NLR scores presented worse outcomes.

ALBI is another appealing clinical index to predict HCC treatment response and survival. It uses two simple live function tests: serum Albumin and Bilirubin exam, to calculate scores and category patients [15]. The utility of ALBI has several advantages. First, it is objective and straightforward. The 2nd, as most HCC cases develop from chronic liver damage, liver functional assessment is of paramount importance in HCC patient management and decision-making. In a systemic review that included 20 studies with 11,365 patients, Geng and his colleagues concluded that higher ALBI was associated with poorer OS [27]. More importantly, they also found that the correlation between the ALBI grade and poor long-term survival was independent of sample size, patient population, follow-up duration, and quality scores [27]. Likewise, a similar conclusion has been reached in a most recent systemic review with 95 studies [9]. In our cohort, however, we identified NLR, not ALBI, as an independent factor in our multivariate model, suggesting that NLR might be superior to ALBI in predicting patient outcomes.

It is clear that multiple clinicopathological factors contribute to and determine the prognostic outcome of HCC patients, such as liver function, pathology grade, treatment plan, etc. Therefore, using a single clinical index to evaluate patients” outcomes is irrelevant. Several index combinations have been reported, such as NLR and platelet-to-lymphocyte [28], psoas muscle mass index with NLR [29], and NLR combined with tumor burden score (TBS) [30]. In this study, NLR combined with ALBI was used for the first time to predict the outcome of HCC. Our work found that these patients with higher NLR-ALBI scores had more extended hospital stays and worse outcomes than those with fewer scores (Table 2; Fig. 2C). Both univariable and multivariable analyses have indicated that the NLR-ALBI score was significantly associated with the OS of HCC patients (Table 3). ROC analysis revealed larger AUC (0.679, 95%CI 0.592–0.767) of NLR- ALBI score than these of NLR alone (0.618, 95%CI 0.526–0.710) and ALBI alone (0.533, 95%CI 0.437–0.629), as shown in Fig. 1. One critical factor reflecting the patient’s per-operative status is systematic inflammation status, which can be assessed by NLR. At the same time, ALBI grade is an index for evaluating the per-operative liver function. Therefore, the combination of NLR and ALBI grade will assess and reflect both patients’ inflammation status and liver function, which will be a better prognostic index for patients with HCC.

In summary, we concluded that NLR is a reliable and inexpensive biomarker and should be incorporated into other predictive models to improve prognostication following HCC treatment. The combination of NLR and ALBI showed a better prognostic performance than using NLR or ALBI alone. These findings may help physicians identify high risk HCC patients with poor outcomes and enable them to consider additional treatment plans and closing monitors of these patients. However, our work was limited in that it was a retrospective study with a relatively small sample size from a single medical center. An internal validation, as exampled by Facciorusso A and his colleagues in their work exploring the factors associated with the recurrence of advanced colorectal adenoma after endoscopic resection [31], is also needed to unequivocally confirm the new model’s reliability and potential clinical application [32]. More studies with large sample sizes are warranted to draw definitive conclusions regarding the predictive capabilities of the combination in determining the long-term outcome of HCC patients.

## Data Availability

The datasets generated and/or analyzed during the current study are not publicly available due to patient privacy and security of electronic medical information but are (anonymized) available from the corresponding author on reasonable request.

## List of Abbreviations

HCC: Hepatocellular carcinoma
AUC: Area under curve
OS: Overall survival
NLR: Neutrophil/lymphocyte ratio
ALBI: Albumin/Bilirubin grade
AFP: Alpha-fetoprotein
CTP: Child Turcotte Pugh
BCLC: Barcelona Clinic Liver Cancer
